# A Simple Model for Diagnosis of Maladaptations to Exercise Training

**DOI:** 10.1186/s40798-022-00523-x

**Published:** 2022-11-04

**Authors:** Mikael Flockhart, Lina C. Nilsson, Björn Ekblom, Filip J. Larsen

**Affiliations:** The Swedish School of Sport and Health Sciences, GIH, The Department of Physiology, Nutrition and Biomechanics, 114 33 Stockholm, Sweden

**Keywords:** Exercise, Performance, Testing, Mitochondria, Maladaptations, Physiology, Overreaching

## Abstract

**Background:**

The concept of overreaching and super compensation is widely in use by athletes and coaches seeking to maximize performance and adaptations to exercise training. The physiological aspects of acute fatigue, overreaching and non-functional overreaching are, however, not well understood, and well-defined negative physiological outcomes are missing. Instead, the concept relies heavily on performance outcomes for differentiating between the states. Recent advancements in the field of integrated exercise physiology have associated maladaptations in muscular oxidative function to high loads of exercise training.

**Method:**

Eleven female and male subjects that exercised regularly but did not engage in high-intensity interval training (HIIT) were recruited to a 4-week long training intervention where the responses to different training loads were studied. Highly monitored HIIT sessions were performed on a cycle ergometer in a progressive fashion with the intent to accomplish a training overload. Throughout the intervention, physiological and psychological responses to HIIT were assessed, and the results were used to construct a diagnostic model that could indicate maladaptations during excessive training loads.

**Results:**

We here use mitochondrial function as an early marker of excessive training loads and show the dynamic responses of several physiological and psychological measurements during different training loads. During HIIT, a loss of mitochondrial function was associated with reduced glycolytic, glucoregulatory and heart rate responses and increased ratings of perceived exertion in relation to several physiological measurements. The profile of mood states was highly affected after excessive training loads, whereas performance staled rather than decreased. By implementing five of the most affected and relevant measured parameters in a diagnostic model, we could successfully, and in all the subjects, identify the training loads that lead to maladaptations.

**Conclusions:**

As mitochondrial parameters cannot be assessed without donating a muscle biopsy, this test can be used by coaches and exercise physiologists to monitor adaptation to exercise training for improving performance and optimizing the health benefits of exercise.

*Clinical trial registry number* NCT04753021. Retrospectively registered 2021-02-12.

**Supplementary Information:**

The online version contains supplementary material available at 10.1186/s40798-022-00523-x.

## Key Points

- Loss of mitochondrial function is an early event during excessive exercise training that seems to precede a loss of physical performance
- We here track physiological and psychological measurements during different training loads and describe how they change during the transition into the maladaptive state
- Using our diagnostic test including non-invasive measurements, we were able to successfully identify the exercise training loads that lead to maladaptations

## Introduction

Athletes stress their body to the limit and accumulate acute fatigue and temporarily sacrifice performance during periods with high training loads, i.e. overreaching (OR) [1]. A decrement in performance is often used to confirm OR, and if subsequent recovery improves performance, the OR is considered to be functional (FOR) and a “super compensation” has occurred [2]. If the OR does not increase performance, the OR is considered to be non-functional (NFOR) and can in the long term develop into overtraining syndrome (OT) [3]. The nature of the maladaptations that could occur during heavy training load is debated, but cardiac [4] and muscular parameters, including myopathologies and mitochondrial dysfunction [5, 6], have been suggested to be impaired during NFOR and OT. In addition, we have recently found intrinsic mitochondrial respiration (IMR), aconitase activity and glucose tolerance to be reduced in healthy subjects after excessive high intensity interval training (HIIT) [7]. A reduced mitochondrial quality has also been found in a cohort of elite triathletes and cyclists after intensified training [8]. It is under debate whether or not a loss of performance is at all needed or desired during OR in order to achieve a supercompensation [9]. Indeed, this causality has been questioned with negative effects on VO_2_max and less gain in performance found in triathletes who showed a reduced performance during OR compared to subjects that were less fatigued during OR [10]. Recent work by Bellinger et al. [11] supports the concept that adaptations of muscle oxidative capacity can be deranged after excessive exercise. In a group of highly trained athletes who performed the same amount of training, an impaired performance during OR resulted in an absence of improvement in muscle oxidative capacity and less increase in performance during super compensation compared to subjects that did not show an impaired performance during OR. It should be noted that the near-infrared spectroscopy method used by Bellinger et al. [11] to assess oxidative capacity has previously been validated against mitochondrial respiratory capacity [12], which is related to mitochondrial content and training volume [13]. In the previously mentioned publications [7, 8], the observed reductions in mitochondrial function occurred in parallel to that several markers of mitochondrial content were unchanged or increased, thereby in part possibly rescuing muscle oxidative capacity.

Several functional approaches exist for monitoring training load and identify the transition into OR and OT. These approaches include measurements of physiological as well as psychological parameters: A reduced performance indicates transition into OR [3]. Oxygen uptake (VO_2_) has been reported to increase during submaximal work rate as well as to be reduced during maximal work rate during OR [14], but those effects are not consistently found [15, 16]. Heart rate (HR) has been found to be reduced during maximal work in FOR [17]. Heart rate recovery (HRR) has been shown to be faster after submaximal and maximal exercise during OR [16]. Ratings of perceived exertion (RPE) [18] have been found to increase relative to power output, HR [19] and lactate [20] during OR and OT. The Profile of Mood States questionnaire (POMS) [21] is regarded as a sensitive tool to detect transition into OR and OT early [22–24]. Hand grip strength has been associated with self-reported fatigue [25] and performance [26]. Blood lactate has been found to be reduced in relation to work rate in OR and OT [15, 27], and resting blood glucose to be reduced during periods with high training loads [28]. Although the mentioned parameters have been studied in applied sports settings, few efforts have been made to combine measurements of several parameters to differentiate excessive from optimal training loads. Difficulties lie in that some symptoms of OT and positive adaptations to exercise training, as for example a decrease in blood lactate in response to work rate [29] and an increase in HRR [30], are characteristic in both situations. Furthermore, none of the mentioned studies have managed to segregate acutely fatigued subjects from subjects being in a more severe state of overreaching, and thereby being able to isolate the effect of an increase in training load, from a maladaptive response, for the mentioned parameters. Diagnosis can therefore not easily be made based on a single parameter, and a combination of different measures is often used [15, 24, 31]. In 1998, Urhausen et al. [27] made a promising attempt to use a multidimensional approach to diagnose OT by combining several of the mentioned parameters and later prompted for more efforts to address this matter [32].

**Fig. 1:**
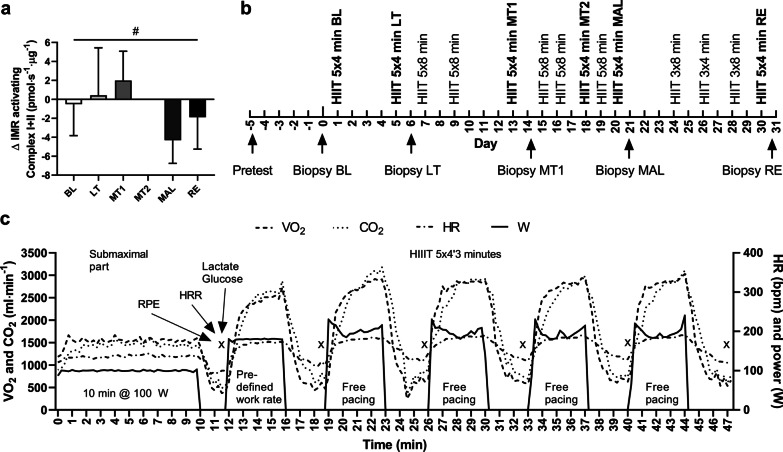
**a** The difference in coupled intrinsic mitochondrial respiration using pyruvate, glutamate, malate, succinate and ADP activating complex I + II in each phase compared to normal respiration (the mean of BL, LT, MT1 and RE measurements), i.e., unaffected by excessive training load. Significant main effect RM one-way ANOVA *p* < 0.05 is marked with #. All values are means ± SD, (n = 11). **b** Study design, **c** a representative HIIT session by a subject showing the acute physiological responses during submaximal warm up at 100 W and at free pacing during HIIT. BL = baseline, LT = light training load, MT1 and MT2 = moderate training load, MAL = maladaptive training load, RE = recovery

We here present data that were collected during a project where we investigated the dose–response effects of HIIT [7]. After administrating the highest dose of HIIT, intrinsic mitochondrial respiration was reduced by 40% (Fig. 1a) and glucose tolerance was impaired. Throughout the study, we closely monitored several physiological and psychological parameters (listed above). We here aim to describe the dynamic response of these parameters and further propose a diagnostic model, using a minimally invasive test, to accurately distinguish between training loads that induce adaptive responses, and excessive training loads that are associated with mitochondrial and metabolic maladaptations (MAL).

## Method

### Experimental Design

To investigate the dose–response effects of HIIT on physiological parameters, we collected a first biopsy at baseline (BL) after 48 h of rest and thereafter adjusted the training load for 4 weeks to be able to detect the first positive adaptations to HIIT after light training load (LT), the cumulative adaptations after a period with moderate training load (MT), after almost daily training that resulted in maladaptations (MAL) and lastly after a period of recovery (RE). From LT and onward, biopsies were collected 14 h post exercise. This time-point was chosen to avoid possible negative acute effects on mitochondrial parameters that have been found immediately after exercise [33, 34]. Also, positive effects of exercise on glucose regulation are expected at this time-point [35, 36]. A schematic of the study design is presented in Fig. 1b. The exercise training load was calculated as minutes of HIIT per week and was at BL 0 min, after LT 36 min, after MT 90 min, after MAL 152 min and after recovery 53 min. Muscle biopsies were collected in fasting state at BL and 14 h after last HIIT session at LT, MT1, MAL and RE for measurement of mitochondrial respiration. To offer higher resolution and track changes of the physiological measurements to secure the transition into MAL, an additional surveilled HIIT session (MT2) was added to the protocol. A detailed method description of biopsy sampling and previously published mitochondrial measurements are presented in Flockhart et al. [7].

### Subject Characteristics

Six female and 5 male subjects were recruited to the intervention. Inclusion criteria were healthy and 18–50 years of age. Exclusion criteria were more than 5 h a week of systematic endurance training, performing regular HIIT, chronic medications or diseases. The female subjects started the intervention at random time-points in their menstrual cycle so the hormonal status would not affect the outcomes. Subject characteristics are presented in Table 1. Subjects were only allowed to perform low intensity training and upper body strength training to maintain their normal training routine. Their own training sessions were planned to not interfere with HIIT sessions and testing during the intervention. Before the start of the intervention, the subjects received information of the study design and possible risks and gave written consent for participation. The study was approved by the local Regional Ethical Review Board in Stockholm and was conducted according to the Declaration of Helsinki.

**Table 1:** Subject characteristics

Subjects	*n*	Age (year)	Weight (kg)	Height (cm)	Lactate threshold (W)	VO_2_max (ml min^−1^)	*W*_max_ (W)
Female	6	25 ± 6	61 ± 6	165 ± 3	156 ± 19	2848 ± 269	233 ± 21
Male	5	31 ± 6	78 ± 8	179 ± 6	202 ± 35	3947 ± 197	303 ± 40
All	11	27 ± 6	69 ± 11	171 ± 9	177 ± 35	3348 ± 617	265 ± 47

All values are measured at the pre-test before the start of the intervention. Values are presented as mean ± SD

### Pretest

A pretest for assessment of baseline physiological characteristics was performed on an SRM ergometer (Schoberer Rad Messtechnik, SRM, Jülich, Germany). The test protocol included a submaximal part with five minutes long stages (starting at 80–100 W and increased with 15–30 W per stage) where the metabolic response to work rate was recorded. Thereafter, a short-step incremental test to fatigue followed for determining VO_2_max (starting at a work rate equivalent to the second lactate threshold and increased with 15–20 W per minute and stage). During the test, VO_2_ and CO_2_ (Oxycon Pro device, Erich Jaeger GmbH, Hoechberg, Germany), lactate and glucose (Biosen C-Line Clinic, EKF-diagnostics, Barleben, Germany), heart rate (Polar Electro OY, Kempele, Finland) were continually sampled and the subjects rated their perceived effort using the Borg RPE 6–20 scale [18]. All equipment was calibrated according to the manufacturer’s instructions, and VO_2_max was defined as the mean of the highest measurements during 40 consecutive seconds.

### HIIT Intervention

In total, 14 HIIT sessions were performed whereof six sessions were performed at the end of each training phase and were extensively monitored with measurements of VO_2_, VCO_2_, capillary blood lactate and glucose, RPEs, HR and HRR. Substrate metabolism was calculated according to the Brouwer equation [37]. The extensively monitored HIIT sessions comprised of five 4-min long intervals intersected by 3 min of passive rest (5 × 4′3). The remaining eight HIIT sessions comprised of five sessions with 5 × 8′3 min, and during RE phase, a taper in the volume of training was applied and three HIIT sessions were reduced by 40% to 3 × 8′3 and 3 × 4′3. The work rate during HIIT corresponds to ~ 90 to 95% of VO_2_max. All 5 × 4′3 HIIT sessions were performed on the SRM ergometer, but some of the remaining HIIT sessions were performed on an additional ergometer (Monark LT2, Monark Exercise AB, Vansbro, Sweden). The 5 × 8′3, 3 × 8′3 and 3 × 4′3 HIIT sessions were performed with measurement of heart rate, RPEs, lactate and glucose to ensure that each subject performed sessions according to their maximal capacity. Before each HIIT session, the subjects reported to the laboratory and filled in the POMS questionnaire and performed measurement of hand grip strength using a dynamometer (Gripen). Each HIIT session was performed with 10 min of warm up at submaximal work rate at 100 W at 70 rpm. We chose to use the same absolute work rate for all subjects as we, and others, have used this approach in different studies. Comparisons of the results between studies can thereby easily be made. Thereafter, two minutes of passive rest followed, with measurement of heart rate recovery and sampling of capillary blood. Then, five intervals, 4 min each, were performed with 3 min of passive rest in between. At the end of each interval, the subjects quickly communicated RPE for their legs and remained seated on the ergometer with their hands on the handlebar and rested for 3 min for heart rate recovery measurement. During the last 30 s of rest, a capillary blood sample was taken and immediately analyzed for glucose and lactate. All HIIT sessions were performed with the subject instructed to perform each session with ambition to produce the highest possible mean power output. To secure a standardized pacing, the subjects were guided to target power output during the first interval of the first session at 117% of the power corresponding to their calculated lactate threshold. This work rate was chosen based on previous trials. Thereafter, free pacing was allowed, and the subjects were able to shift gear and alter cadence and resistance according to their strategy to maximize performance with the time remaining of the interval as the only feedback. The highest mean power from any previous session was then used as target work rate during the first interval at next HIIT session. The subjects were blinded to their performance throughout the study and were monitored during all tests and HIIT sessions by personnel with extensive experience in training and testing methodology. See Fig. 1c for a representative HIIT session.

### Nutritional Standardization

Prior to the first biopsy sampling at baseline, the subjects had consumed a controlled diet during the previous day and then remained fasting until biopsy sampling in the morning. The evening meal was provided by the research team and contained 74 g of carbohydrates, 38 g of protein and 26 g of fat and were the same for all subjects. The remaining meals were individually chosen (breakfast, lunch and additional snacks) by the subjects and had a balanced composition of carbohydrates, fat and protein. Later during the intervention when HIIT sessions were performed in the late afternoon, a recovery drink was consumed by the subjects containing 1 g kg^−1^ bw of carbohydrates and 0.25 g kg^−1^ bw of protein immediately after HIIT and the evening meal was consumed 2 h post HIIT. During the recovery phase when the workload of three HIIT sessions were reduced, the recovery drink was reduced by 50%. The timing of meals, HIIT sessions and biopsies were repeated throughout the intervention and strictly scheduled for each subject. During the rest of the intervention period, the subjects were free living and consumed their own diet of choice and were guided to maintain a balanced energy intake.

### Establishing a Tool to Diagnose Maladaptations to Exercise Training

We wanted to establish a simple model using physiological and psychological variables that could indicate the development of maladaptations. Considering which easily accessible parameters that were most affected during the MAL phase, we chose to incorporate the fatigue parameter from the POMS questionnaire, the maximal values of HR, blood lactate and glucose at the end of HIIT sessions and mean RPEs during HIIT. A baseline for each individual and parameter during “normal” exposure to training load was determined by calculating the individual mean from measurements in BL, LT, MT1 and RE (where measurements were unaffected by maladaptive training loads). To determine if the training load during MAL resulted in markedly higher (POMS fatigue and RPEs) or lower values (Lactate_end_, Glucose_end_ and HR_max_) than normal values, cut-offs (Table 2a) were established as half of the difference between the group means during MAL and group means during normal training load. If a subject during any training load deviated more from their individual normal value than the cut-off value, this was interpreted as indication of MAL and was given a positive score of 1. If the criteria was not fulfilled, the score was 0. Cut-off values are presented in Table 2. To assess whether changes in mitochondrial respiration were related to the number of accumulated scores in each phase, normal respiration was calculated as the mean of BL, LT, MT1 and RE measurements for each subject and the difference in each phase compared to normal values was correlated to the number of scores for each subject.

**Table 2:** (a) Cut-off values and number of positive scores for the selected parameters; POMS_fatigue_ (score), HR_max_ (bpm), glucose_end_ and lactate_end_ (mmol L^−1^) and Borg RPE_mean_ (scale 6–20) during HIIT. (b) A summary of subjects having n of 5 scores during each phase of training load. During MAL, all 11 subjects achieved more 3 or more scores, whereas non had 3 or more scores in BL, LT, MT1 and RE

**a** Number of subjects achieving a positive score for each parameter										
Diagnostic parameter	Mean normal	Mean MAL	Mean diff	Cut-off value	BL	LT	MT1	MT2	MAL	RE
POMS_fatigue_	42.6	51.9	+ 9.3	+ 4.7	0	2	1	3	9	1
HR_max_	183.6	178.5	− 5.1	− 2.5	1	0	5	5	10	2
Glucose_end_	6.11	4.68	− 1.43	− 0.71	2	2	0	7	9	0
Lactate_end_	13.28	12.03	− 1.25	− 0.62	4	3	7	6	7	1
RPE_mean_	17.5	18.5	+ 1.0	+ 0.5	0	0	0	5	9	9

**b** Number of subjects achieving *n* of 5 scores in all phases						
Scores	BL	LT	MT1	MT2	MAL	RE
≥ 1 of 5	5	6	9	9	11	9
≥ 2 of 5	2	1	4	6	11	4
≥ 3 of 5	0	0	0	6	11	0
≥ 4 of 5	0	0	0	4	6	0
5 of 5	0	0	0	1	5	0

*n* = 11 in all phases

*BL* baseline, *LT* light training load, *MT1 and MT2* moderate training load, *MAL* maladaptive training load, *RE* recovery

### Mitochondrial Measurements

Muscle biopsies were collected from Vastus Lateralis at time-points BL, LT, MT1, MAL and RE. Mitochondria were isolated from tissue, and respirometry was performed on the resulting mitochondrial suspension using an Oxygraph-2 k (Oroboros Instruments Corporation, Innsbruck, Austria). The highest values during ADP-stimulated respiration activating complex I + II using pyruvate, glutamate, malate, and succinate were related to the protein content in the mitochondrial suspension measured with a Pierce 660 nm protein assay (Thermo Fisher Scientific). In Fig. 1a, the respiration in each phase is related to normal respiration, calculated as the mean of measurements in BL, LT, MT1 and RE-phase. Further methodological details are available in Flockhart et al. [7].

### Data Analysis

During submaximal exercise at 100 W for 10 min, the VO_2_, VCO_2_ and heart rate measurements for the last 7 min were used. The data collected during HIIT are expressed as either session means calculated from each interval, the session mean calculated from max values during each interval or the session mean calculated from values collected during/after the last interval. Power output and heart rate were calculated from the full interval length, and for VO_2_, the highest consecutive 120 s of VO_2_ measurements during the plateau of each interval was used. The HR_max_ value during HIIT refers to the highest 30 s mean anytime during a session. Heart rate recovery was calculated as the difference from the highest 30 s during submaximal work rate to each defined time-point. During HIIT, heart rate recovery was calculated after each interval using the difference from the highest 30 s to each defined time-point. A session-mean was then calculated. End lactate and glucose values refer to the values sampled at the end of HIIT sessions. POMS scores were analyzed in POMS:EDITS (MacPOMS: DataMedic AB & Melebo AB, San Diego, USA). Total mood disturbance (TMD) is calculated from Tension + Depression + Anger + Fatigue + Confusion − Vigor) and the Energy Index (EI) from Vigor to Fatigue. Mitochondrial analyses were performed in DatLab 5.2 software (Oroboros, Paar, Graz, Austria).

### Statistics

Normality was checked using a Shapiro–Wilk test, and repeated measures one-way ANOVA or Freidman’s test was used to detect a main effect. Correlations were calculated using Pearson’s correlation coefficient (GraphPad prism 8.3.1). A *p*-value of < 0.05 was considered significant. Results are expressed as means ± standard deviation (SD) unless otherwise stated.

## Results

### Performance, Oxygen Uptake, HR and HRR

**Fig. 2:**
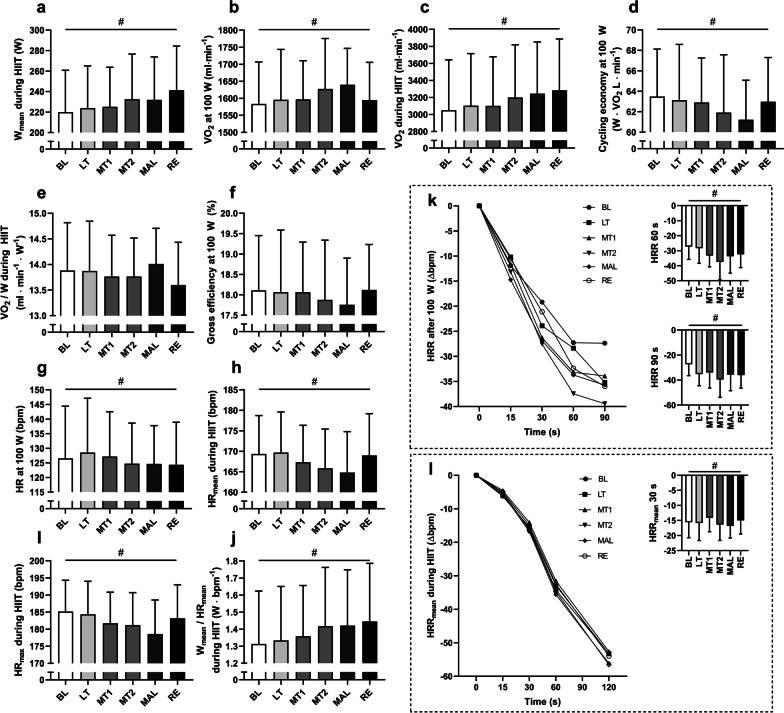
**a** Power output during HIIT, **b** VO_2_ at submaximal work rate, **c** VO_2_ during HIIT, **d** cycling economy at submaximal work rate, **e** VO_2_ per W during HIIT, **f** gross efficiency at submaximal work rate, **g** HR at submaximal work rate, **h** mean HR during HIIT, **i** max HR during HIIT, **j** power output per HR during HIIT, **k** mean HRR after submaximal work rate and **l** mean HRR after HIIT. HRRs with a significant main effect is highlighted in separate graphs. Significant main effect RM one-way ANOVA *p* < 0.05 is marked with #. All values are means ± SD, (*n* = 11). Figure g has previously been published [7]. BL = baseline, LT = light training load, MT1 and MT2 = moderate training load, MAL = maladaptive training load, RE = recovery

Power output during HIIT (Fig. 2a) increased continuously during the intervention except during MAL when performance staled. Oxygen uptake showed the highest values during MAL at submaximal workload (Fig. 2b) and increased continually during HIIT (Fig. 2c). Cycling efficiency (Fig. 2d) at submaximal work rate was at its lowest during MAL. Likewise, the VO_2mean_/*W* ratio during HIIT (Fig. 2e) was at its highest during MAL. Gross efficiency at submaximal work rate (Fig. 2f) was, however, not significantly affected but showed the lowest values during MAL. HR during submaximal workload (Fig. 2g) was not reduced during MAL. During HIIT, mean HR (Fig. 2h) and maximal HR (Fig. 2i) showed substantially suppressed values during MAL. The *W*_mean_/HR_mean_ ratio during HIIT (Fig. 2j) increased throughout the intervention. HRR was affected by training load at 60 and 90 s after submaximal work (Fig. 2k) rate and at 30 s after HIIT in a dynamic fashion (Fig. 2l). In all, the changes in HRR were small and post hoc tests revealed that only HRR_30s_ during HIIT was decreased between MT1 and MAL.

### Blood Lactate and Glucose

**Fig. 3:**
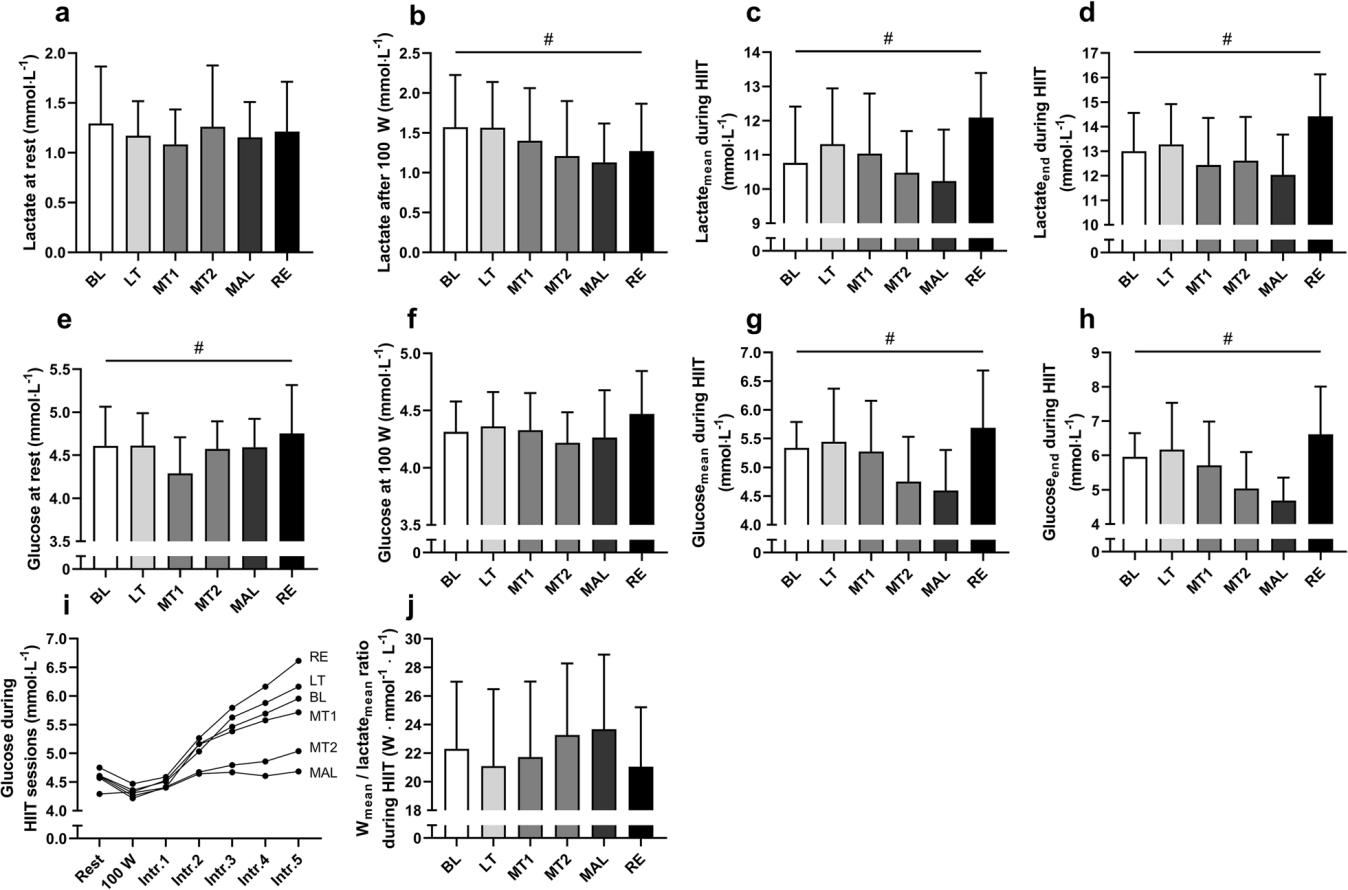
**a** Lactate at rest, **b** lactate after submaximal work rate, **c** mean lactate during HIIT, **d** lactate at end of HIIT, **e** glucose at rest, **f** glucose after submaximal work rate, **g** mean glucose during HIIT, **h** glucose at end of HIIT sessions, (**i**) glucoregulatory response during intervals, **j** power output per lactate during HIIT. Significant main effect RM one-way ANOVA *p* < 0.05 is marked with #. All values are means ± SD with exception of (**i**), (*n* = 11). Figures d, f and h have previously been published [7]. BL = baseline, LT = light training load, MT1 and MT2 = moderate training load, MAL = maladaptive training load, RE = recovery

Lactate was unaltered during rest (Fig. 3a) but showed the lowest values during submaximal work rate during MAL (Fig. 3b). Mean lactate (Fig. 3c) as well as lactate at the end of HIIT (Fig. 3d) followed the same pattern but, in addition, increased sharply after recovery. Resting glucose (Fig. 3e) and glucose at submaximal work rate (Fig. 3f) were not reduced during MAL but was highly affected during HIIT, showing the lowest mean values (Fig. 3g) as well as values at the end of HIIT (Fig. 3h) during MAL. The glucoregulatory response during HIIT sessions is presented in Fig. 3I. The ratio between mean power output and lactate during HIIT (Fig. 3j) was not affected.

### Mood States and Central and Local Fatigue

**Fig. 4:**
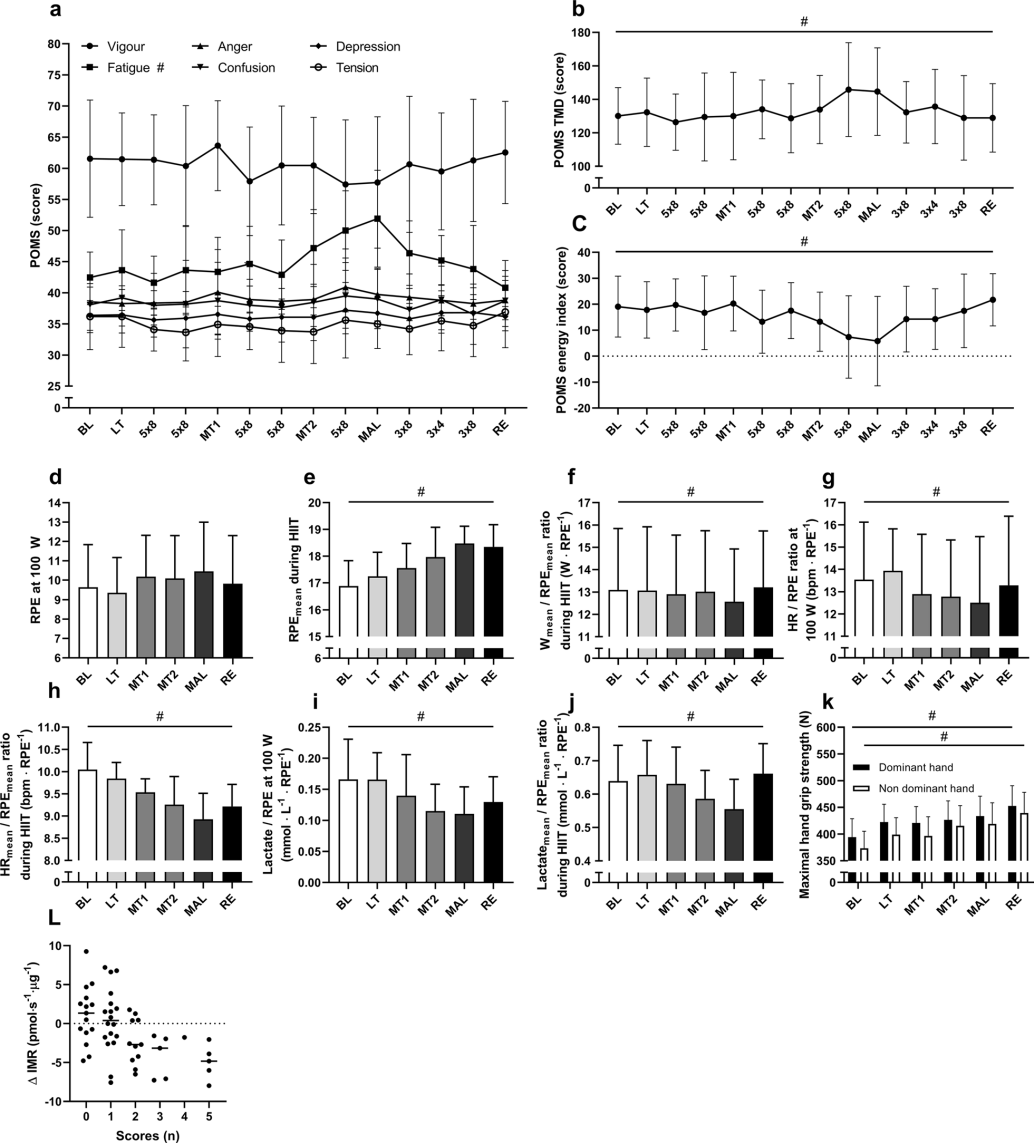
**a** POMS scores during the whole intervention, **b** total mood disturbance, **c** the energy index (vigor–fatigue), **d** RPEs at submaximal work rate, **e** RPEs during HIIT, **f** power output per RPE during HIIT, **g** HR per RPE during submaximal work rate, **h** HR per RPE during HIIT, **i** lactate per RPE during submaximal work rate, **j** lactate per RPE during HIIT and **k** maximal hand grip strength. Significant main effect RM one-way ANOVA *p* < 0.05 is marked with #. All values are means ± SD, (*n* = 11). **l** The number of scores achieved in each phase using our diagnostic test and the difference in intrinsic mitochondrial respiration activating complex I + II in each phase compared to normal respiration (the mean of BL, LT, MT1 and RE measurements). Each subject and situation is represented by a dot. BL = baseline, LT = light training load, MT1 and MT2 = moderate training load, MAL = maladaptive training load, RE = recovery. 5 × 8, 3 × 8 and 3 × 4 = HIIT training sessions

The full results of profile of mood states are presented in Fig. 4a. Fatigue was the only mood state that was affected throughout the intervention with a sharp increase during MAL. Total mood disturbance (TMD) (Fig. 4b) peaked during MAL, and the calculated energy index (Fig. 4c) was at its lowest during MAL. During exercise, RPEs at submaximal work rate (Fig. 4d) were unaltered but during HIIT (Fig. 4e), and they displayed a steady increase with training load and were at their highest during MAL and RE. The *W*_mean_/RPE_mean_ ratio during HIIT (Fig. 4f) showed a decline during MAL but otherwise remained stable during the whole intervention. The HR/RPE ratio at submaximal work rate (Fig. 4g) and during HIIT (Fig. 4h) showed the lowest values during MAL. Likewise, the lactate/RPE ratio was at its lowest during MAL during both submaximal work rate (Fig. 4i) and during HIIT (Fig. 4J). Hand grip strength (Fig. 4k) increased steadily during the intervention.

### A Diagnostic Tool for Identifying Maladaptations to Exercise Training

By using our diagnostic model, we found that exceeding the cut-off values on three or more of the five parameters had the highest power to identify a subject as having maladaptations (Table 2b). Using this approach, all 11 subjects were correctly identified as having maladaptations during the MAL phase, whereas none were identified as having maladaptations during the MT1 phase. The number of achieved scores was further associated with changes in mitochondrial respiration (Fig. 4l) (Pearson *r* = − 0.50, *p* < 0.001). An example of calculations of a subject is available in Additional File 1: S1.

## Discussion

Here, we present data on several physiological and psychological parameters that are affected during increased training load. A selection of the most affected and easily accessible parameters has been put into a model to offer a tool for early detection of MAL.

### Physiological Responses to HIIT

We observed a steady increase in performance throughout the intervention except during the maladaptive phase. A decrease in performance is often used as a main criterion for diagnostics of OR, NFOR and OT, with the time for restoration of performance separating the different states [38, 39]. Therefore, the dramatic loss of mitochondrial function did not translate into a corresponding loss of performance. Measurement of performance appears therefore not to be a sensitive marker for confirming the observed type of metabolic maladaptation. If confirmation of OR and differentiation between FOR and NFOR is based primarily on performance, and can be made first after recovery, negative outcomes can manifest unnoticed. A plausible reason for why performance was maintained during MAL can be the observed increase in VO_2_. This indicates that oxygen delivery was not compromised during MAL. However, we observed a reduction in cycling economy during MAL. Changes in cycling economy can be affected by alterations of mitochondrial properties as an adaptive response to favor high energy production through a bypass of mitochondrial complex I, as has been shown to occur during high intensity exercise [40]. We interpret the excessive VO_2_-response during exercise as a favorable adaptation to the high intensity training. During HIIT, HR decreased during MAL. A decreased HR at a fixed work rate can be a symptom of parasympathetic overtraining [41], and it has been demonstrated in triathletes during FOR that maximal stroke volume, cardiac output and peripheral oxygen extraction can be negatively affected during a maximal test together with increased fatigue (POMS), reduced HR, VO_2_max, lactate, and power output [42]. Oxygen uptake was maintained in our subjects, and although the produced power output per heartbeat increased during MAL, it was even higher after recovery when performance peaked. We also measured HRR. A more rapid HRR is associated with improved athletic capacity, and subjects with an increased HRR have shown better adaptation to HIIT than subjects showing a continuous decrease in HRR at the end of OR [43]. In agreement with Lamberts et al. [43], we observed the fastest HRR after submaximal work rate during MT2. Thereafter, HRR decreased during MAL and RE. HRR was therefore not a sensitive parameter for the transition into MAL.

Blood lactate and especially glucose were the physiological parameters showing the most robust changes in relation to training load. We do not attribute the suppressed values of lactate to energy unavailability as we have previously shown that glycogen stores in our subjects increased with training load rather than were compromised [7]. The low values of lactate during submaximal work rate as well as during HIIT could be interpreted as positive adaptations if the reduced peak values of lactate is not accounted for. In 2001, Bosquet et al. [29] proposed a model where submaximal lactate was adjusted to changes in peak lactate. Changes in upward or downward shift could thereby be separated from leftward or rightward shift of the lactate curve with intent to discriminate between reduced maximal glycolytic capacity and true positive adaptations. A less investigated indicator of OR is the glucoregulatory response during exercise. We here found retained glucose at rest and after submaximal exercise, but during HIIT, glucose decreased during MAL. The low blood glucose values observed are likely not explained by an increased glucose uptake by the working muscles since we in the same training phase observed a reduced glucose uptake during an oral glucose tolerance test performed on these subjects [7]. The assessment of lactate and especially glucose alterations appear therefore to constitute sensitive and stable measurements for detecting maladaptations.

### Central Fatigue, Perceived Exertion and Mood States

Decrement in performance during overreaching has been attributed to an increased central fatigue caused by glycogen depletion [44] and elevated plasma FFA levels, resulting in increased serotonin in the brain [45]. In contrast to these hypotheses, we observed normal muscle glycogen and plasma FFA in the fasted state [7]. We also measured hand grip strength as a possible marker of neuromuscular fatigue but found it to increase during the full intervention period. During exercise, RPEs can be used to detect altered exertion in relation to a second quantifiable parameter [46]. Decreases in HR/RPE and performance/RPE ratios have been proposed to be associated with OR and also be inversely correlated to increased performance after an OR/taper-period in cyclists [19]. A decreased lactate/RPE ratio has been observed in cyclists during OT [20]. In support of previous research, we also found consistent alterations for HR, lactate, power output and RPE ratios during submaximal work rate and during HIIT. This indicates that accumulated fatigue indeed affected performance and that RPE ratios can offer high sensitivity for detecting maladaptations. Using the POMS questionnaire, we also found the fatigue parameter and the energy index to be highly affected during the intervention. It has previously been shown that POMS can be used to successfully separate acutely fatigued subjects from FOR-subjects [10]. Interestingly, in that study the subjects that improved performance the most after an overload training phase and a taper were those that rated less increase in fatigue and had the highest energy index during high training loads. Measurements of mood states and RPEs should therefore be implemented in a multidimensional approach for detection of maladaptations.

### A Diagnostic Tool for Monitoring Transition into Maladaptive Exercise Training Loads

In our model, we chose to include the fatigue parameter from the POMS questionnaire, RPEs, lactate and glucose at the last interval and maximal HR during HIIT. These parameters are easy to assess, and glucose and lactate sampled at the end of each session in most cases represent peak values. It should be noted that low levels of lactate by itself do not indicate MAL but do so with a concomitant increase in RPEs. Using the incorporated parameters in isolation can therefore cause misleading interpretations. This emphasizes the need of a multidimensional test for diagnosis. The parameters that showed the strongest association with maladaptations in our study were in part also identified by Urhausen et al. [27]. They followed 17 male endurance athletes over 19 months. 13 of 15 indices of OT could be confirmed by subjects fulfilling two of following criteria: a reduction of maximal lactate and/or HF, a reduced performance and a reduced “capacity to act” according to the Nitsch scale. Unlike the method used here, Urhausen et al. did not have a physiological parameter defining the OT-state but instead confirmed OT in subjects when they were exposed to heavy training loads or experienced pronounced fatigue and staleness. As cut-off values, the lowest measurements for each parameter and individual during exposure to normal training loads were used. In this study, the responses during normal training loads were defined as all training loads where maladaptations did not manifest. Other approaches were also tested, including restricting the inclusion of normal (baseline) values to measurements in MT1, and to construct cut-offs based solely on individual measurements. These approaches did, however, result in less stable baselines, and in some cases too high or low cut-off values. We therefore believe that the suggested approach can be used with confidence, and that both baselines and, if chosen, individual cut-off values can be further stabilized over time when the model is implemented by athletes and coaches. Future adjustments of our cut-off values and validation in different populations and sport specific situations are needed. Establishing baseline values for each individual cannot be circumvented because of the large between-individual differences on each of the assessed parameters.

### Limitations of the Study and Suggestions for Future Research

In contrast to our one-dimensional study design using solely HIIT, competitive athletes normally perform a mixture of different exercises which induces stress in different ways. Therefore, the threshold for a loss of mitochondrial function and resulting outcomes may be further investigated in other groups of athletes inducing stress with different training regimens. It should also be investigated whether further improvements in performance may be achieved by implementing our diagnostic model and adjust training load with intention to avoid maladaptations. Further research should also adopt a multidimensional approach with additional assessment of recovery, for example by monitoring sleep. We did not find any sex differences for the mitochondrial, physiological, or psychological responses to different training loads. However, future investigations should consider that possible sex differences could exist.

## Conclusions

For optimizing training- and health outcomes, we suggest a multidimensional approach for monitoring exercise training load. For continuous use of the proposed diagnostic model in athletes' normal training, the selected parameters could be measured during any test or session performed at high intensity and with strict standardization. The selected parameters would likely respond to sessions limited by voluntary effort (as used in the present study) as well as during a fixed work rate at 90–95% of VO_2_max. By using data collected during training sessions, the need for a specific test is avoided. We therefore recommend our selected parameters to be implemented on an individual level and used for long-term follow-up.

## Supplementary Information

**Additional file 1**. Calculation of scores for one subject.

## Data Availability

The datasets used and/or analyzed during the current study are available from the corresponding author on reasonable request.
