# Supplementary material for: A Simple Model for Diagnosis of Maladaptations to Exercise Training
Source: Sports Med Open. 2022 Nov 4;8:136. doi: 10.1186/s40798-022-00523-x (PMC9636365; doi:10.1186/s40798-022-00523-x)
Supplement: Supplementary file 1 — Additional file 1. Calculation of scores for one subject. [file 40798_2022_523_MOESM1_ESM.docx]

Supplement material Sports Medicine Open

**A simple model for diagnosis of maladaptations to exercise training**

**Flockhart M^1*^, Nilsson LC^1^, Ekblom B^1^, Larsen FJ^1*^**

^1^The Swedish School of Sport and Health Sciences, GIH; The institution for physiology, nutrition and biomechanics; Stockholm 114 33 Stockholm; Sweden.

^*^Correspondence: [mikael.flockhart@gih.se](mailto:mikael.flockhart@gih.se) (M.F), [filip.larsen@gih.se](mailto:filip.larsen@gih.se) (FJ.L)

| Diagnostic parameter | Individual normal values | Cut-off value | Individual cut-off value | BL | LT | MT1 | MT2 | MAL | RE |
| --- | --- | --- | --- | --- | --- | --- | --- | --- | --- |
| POMS fatigue | 37.5 | +4.7 | >42.2 | 37 (0) | 37 (0) | 37 (0) | 42 (0) | 46 (**1**) | 39 (0) |
| Max heart rate | 170.0 | -2.5 | <167.5 | 171 (0) | 170 (0) | 172 (0) | 168 (0) | 165 (**1**) | 167 (**1**) |
| End glucose | 5.3 | -0.71 | <4.54 | 5.08 (0) | 5.59 (0) | 4.59 (0) | 4,74 (0) | 4,52 (**1**) | 5.75 (0) |
| End lactate | 12.4 | -0.62 | <11.78 | 11.84 (0) | 11.77 (**1**) | 10.79 (**1**) | 15.03 (0) | 11.50 (**1**) | 15.22 (0) |
| Mean RPE | 16.8 | +0.5 | >17.3 | 16 (0) | 16.4 (0) | 16.6 (0) | 17.8 (**1**) | 18.2 (**1**) | 18.2 (**1**) |
|  |  |  |  |  |  |  |  |  |  |
| SUM of scores |  | |  | **0** | **1** | **1** | **1** | **5** | **2** |

**Supplement 1** calculation of scores for one subject for the selected parameters; POMS_fatigue_ (score), HR_max_ (bpm), glucose_end_ and lactate_end_ (mmol · L^-1^) and Borg RPE_mean_ (scale 6-20) during HIIT. Normal values are the mean of measurements in BL, LT, MT1 and RE. Cut-off values are half the difference between normal values and values during NFOR for the whole group of subjects. The individual cut-off values are the individual normal values + or – the cut-off values. If a subject had a higher (POMS_fatigue_ and mean RPE_mean_) or lower (HR_max_, glucose_end_ and lactate_end_) value in each phase than the individual cut-off value, a score of 1 was noted for that parameter. The sum of scores for the five parameters determines the result of the diagnostic test. BL = baseline, LT = light training load, MT1 and MT2 = moderate training load, MAL = maladaptive training load, RE = recovery.
